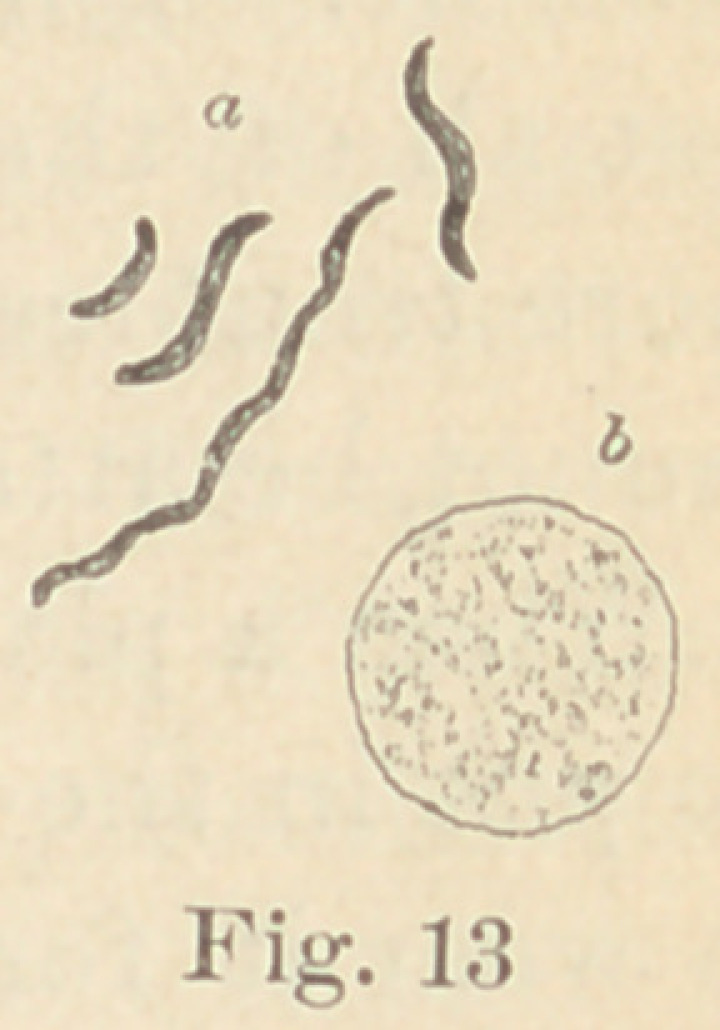# Biological Studies on the Fungi of the Human Mouth

**Published:** 1885-06

**Authors:** W. D. Miller

**Affiliations:** Berlin


### Independent Practitioner.

VOL. VI. JUNE, 1885. No. 6.

### Original Communications.

### BIOLOGICAL STUDIES ON THE FUNGI OF THE HUMAN MOUTH.

BY PROF. DR. W. D. MILLER, BERLIN.

(Concluded from page 232.)

By use of the methods described I have isolated twenty-two different fungi from the secretions or deposits of the human mouth, and have endeavored to determine, as far as possible, their separate peculiarities of growth, physiological action, etc. It will, however, at once suggest itself to every one, that a thorough study of twenty-two different fungi involves an enormous amount of labor, and might constitute almost a life task for one experimenter. The task is, moreover, rendered still more difficult by reason of the fact that many of these fungi show differences of action when cultivated in different media, rendering the number of experiments necessary to come to a definite conclusion doubly great. I shall, therefore, not attempt to present an exhaustive treatment of the subject, but rather an introduction, hoping, at the same time, to establish some points which may be of use in bringing about a clearer understanding of the factors involved in the production of dental caries.

Regarding the first point to be considered—the morphology of the fungi—it is not at all necessary to enter into a minute description of all the different forms here presented. The figures will give a sufficiently clear idea of their diversity, and the appearance of their colonies under a low power. For the rest, suffice it to say that ten of them are micro- or diplococci, five are bacteria and six bacilli. Some show more than one form of development. It would, however, lead us too far from our subject to discuss this fact here.

In liquid media, three grow out into long leptothrix, forming bundles or meshes of intertwining uni- or multicelluar threads, while one develops into spirilli; eight are motile, fourteen are non-motile, while three only have been seen to form spores. The others multiply by division alone.

With reference to the latter point, however, I have not made examinations sufficiently careful or extensive to be able to speak decidedly. Eight liquify nutritive gelatine, one converts it into a paste, thirteen leave it unchanged. On agar-agar, the differences of growth are not sufficiently pronounced to deserve particular mention. In gelatine, the microscopic appearance of the colonies of a sufficient number of these fungi, is shown in the figures (b). It will be seen that the appearance of the colonies forms a much safer means of differentiation than the morphological characteristics of the fungi, it being very seldom that, in growing, two fungi present exactly the same appearance. An exception is, however, presented by 6 and 7, which to the naked eye and under the microscope grow on gelatine exactly alike; moreover, on potato, white of egg, blood-serum, agar-agar and milk, their effect is identical. One, however, produces a yellow coloring matter, the other not, and thereby they are easily distinguished. The others may all be readily distinguished by their growth on potato.

Fig. 1

In relation to oxygen, they show great differences. Ten are strictly aerobian ; i. e. they grow only where the air has free access. Four are not strictly aerobian; i. e. they propagate also when the atmospheric air is excluded, though not so rapidly. Eight grow equally well with or without access of air. Sixteen produce an acid reaction in a solution of beef extract, peptone and sugar. Four produce an alkaline reaction without the appearance of bad smelling products, and appear to leave the solution neutral. With regard to the six, however, the results were not satisfactory, sometimes the reaction being acid, at other times neutral or alkaline, depending somewhat upon the material used for the cultures.

Fig. 2

Some which produce an acid reaction in fermentable solutions, give rise to an alkaline reaction in non-fermentable solutions. The acid produced is probably, in all, or in nearly all these cases, lactic acid. This fact I established for No. 1 by chemical analysis, for No. 2 by forming the zinc salt and crystallizing, for No. 5 by the color test.* In the other cases the acid was not determined. Thirteen were repeatedly cultivated on potato. Of these, five grew rapidly, one in particular covering the whole surface of the section in forty-eight hours, and completely liquifying it to a depth of one to two mm., the liquified mass flowing off at the sides; the others develop very slowly, and attain only a limited growth. I am not able to say whether any of them possesses a diastatic action. It is, however, highly probable. Fifteen were cultivated on boiled white of egg. Four grew very rapidly, No. 19 (see fig. 13) in particular, in from two to four days, converting the egg into a semi-transparent, pasty mass, which gradually disappeared. In the first two days large quantities of sulphuretted hydrogen are developed; later, ammonia. Seven grew slowly on the white of egg, and four scarcely at all. The nourishment of the fungi naturally takes place at the expense of the albumen of the egg, which is converted into a soluble variety by the peptonizing action of the fungus. In two cases the presence of peptone could be detected in the dissolved mass after separation from the albumen, by the biuret reaction, the organisms producing more peptone than they needed for their own consumption.

Fig. 3

Some of them produce in fermentable solutions considerable quantities of gas. If a glass bulb, with a fine stem drawn out to a point, be filled with milk inoculated with No. 3, (see fig. 2), otherwise sterile, and kept at blood temperature, in twenty-four hours so much gas will be generated that on breaking off the point the whole contents of the bulb will be ejected with considerable force. The same effect

Fig. 4

*Two drops carbolic acid, one drop chloride of iron, twenty ccm. water, produce a violet color which becomes yellow on the addition of lactic acid, even in very dilute form. I am not prepared to say that this is an absolutely sure test for lactic acid. It is the test used by Prof. Ewald and others, for detecting lactic acid in the stomach, and is considered by them to be decisive. Of course the culture material itself must not give this reaction. Beef extract, for example, cannot be used, as it already contains lactic acid. A few other substances also give this reaction, but none, I believe, which are likely to be produced in these cultures.

may sometimes be produced, though not so markedly, when non-fermentable solutions are used. We may expect a similar action to take place when we seal up a dead pulp in a tooth, not only the gas itself escaping through the apical foramen, but, if its exit is hindered, ultimately forcing particles of the decomposing pulp through with it. The question suggests itself, whether certain configurations seen in carious dentine may not owe their origin in part to the pressure of gas.

Fig. 5

Four produce coloring matter, Nos. 5 and 7 (figures 4 and 5), in gelatine cultures some days' old, forming brick-yellow masses, such as may be seen occasionally on the buccal surface of teeth which are not kept well cleaned.

On potato they appear bright yellow. Nos. 10 and 13 give the gelatine for a space one cm. in diameter around the colony, a grass-green tinge. I doubt very much whether either of these organisms has anything to do with the production of green stain, all my attempts to isolate a chromogenic fungus directly from green stain having thus far failed. Cultures of some of these fungi were made on dentine and enamel. Sections of dentine, when decalcified neutralized and soaked in saliva and sugar, formed, when kept in a perfect damp cell, a medium on which a considerable development took place, microtome sections of the dentine after two weeks showing a destruction of substance at the point of inoculation.

Fig. 6

On sections of normal dentine, the fungi in some cases appeared to maintain an existence until the organic matter exposed upon the surface of the section was consumed, after which the development ceased, while normal enamel, as might have been expected, formed about as good a culture substratum as glass or porcelain.

A description of the cultures in milk, blood-serum, etc., is not necessary for our present purpose. Also, experiments on animals have been made in too limited a number to lead to accurate results. It is very plain, however, that a study of the pathogenic character of twenty-two fungi is out of the question. No. 19, which possesses peculiar interest on account of its similarity to the cholera-bacillus, was tested on mice, guinea-pigs and rabbits. A small quantity from a pure culture injected into

Fig. 7

the abdominal cavity of mice, almost invariably caused death in a few hours. Guinea-pjgs and rabbits have thus far shown themselves proof against it, even when large quantities were injected into the duodenum (the *ductus choledochus* not being ligated.) Experiments were made with a number of antiseptics, in adition to those reported in this journal (Vol. V. page 283.) Arsenious acid, contrary to the repeated statements of one of our journals, possesses an antiseptic power at least half as great as that of carbolic acid, and about twenty-five times greater than absolute alcohol. Chlorate of potassium, on the other hand, possesses scarcely any available power whatever. Peroxide of hydrogen proved to be particularly active. These experiments are not yet completed, and will therefore be given in a separate paper.

Fig. 8

The following practical conclusions appear to follow from the experiments above recorded:

1. A great majority of the fungi found in the human mouth are capable of producing acid from cane or grape sugar, and it is probable that, *with very few exceptions,* all can, when the proper conditions are presented to them. In nearly all cases which have been examined with special reference to this question, the acid has appeared to be lactic. The acetic acid fermentation, which cannot go on at temperatures above 35° C. (Fluegge), is out of the question in the human mouth, nor is there, as yet, any proof of the presence of more than minute traces of butyric acid.

2. In non-fermentable substances, the reaction will be found either neutral or alkaline, in some cases considerable quantities of ammonia and sulphuretted hydrogen being produced. If, therefore, a decomposing pulp is sealed up in a tooth, its reaction cannot be acid, and caries cannot take place either in the pulp chamber or root canals.

3. Of considerable interest is the fact that the same fungus may produce an acid reaction in one substratum, and an alkaline in another. If, for example, No. 19 (Fig. 13) be cultivated in certain neutral, non-fermentable substances, an alkaline reaction will appear. If, then, sugar be added, the reaction will in a few hours change to acid. In such a case we undoubtedly have two distinct processes going on; first, the nutrition of the organ-

Fig. 9

ism accompanied by the appearance of alkaline products, and secondly, its fermentive action accompanied by acid products. Ordinarily, the latter so outweigh the former that the resultant reaction will be acid. This is, however, by no means necessarily the case. On the other hand, conditions may readily be produced under which the resultant reaction will be neutral or

Fig. 10

alkaline, especially in the human mouth, where so many different fungi and so various conditions are present. In such a case, the result would be to put a temporary check upon the advance of the decalcifying process; in other words, upon the caries itself. In the case of particularly foul-mouthed persons, the foulness itself may become a preventive of caries.

4. The possession of a peptonizing action by a large number of these fungi, readily accounts for the solution of the decalcified dentine.*

5. Any one of these fungi which can produce acid by fermentation of carbohydrates, or can dissolve the decalcified dentine, may aid in the production of caries, while any one which combines both these properties, as many of them do, may alone bring about the phenomenon of dental carries. A solution of the dentine or enamel,

Fig. 11

without previous decalcification, cannot take place. The fact which I have so often affirmed, and which was denied by Milles and Underwood, that one continually meets with large tracts of softened, non-infected dentine, has been completely confirmed by Arkovy and Matrai. They say, "the invasion extends, however, only to a certain depth, and only isolated tubules show a deeper in-

*Not a little confusion has been introduced by attempted artificial definitions of putrefication and fermentation. The idea that every change in nitrogenous organic substances must be of the nature of putrefaction, is particularly misleading. A ferment of the nature of pepsine, which dissolves coagulated albumen, is widely distributed among the fungi of fermentation, as well as putrefaction, and the schizomycetes in general require nitrogenous substances in some shape, for their nutrition. The dissolution of the organic portion of dentine is by no means dependent upon the presence of putrefactive organism, but may be accomplished equally well by fermentation. As stated in previous papers, I never found a putrefactive organism in the deeper portions of carious dentine. Moreover, the acid reaction of carious dentine is highly unfavorable to the development of such organisms. I intend to repeat and extend my experiments on this point. The presence of putrefactive organisms, while it would accelerate the second stage of caries, could only retard the first vasion, sometimes to twice the depth, and reach the border of the normal dentine," the whole territory between the isolated tubules being free from invasion.

Fig. 12

6. The comparative or complete independence of many of these organisms of the free access of air, renders their propagation within the dentine, or under fillings where softened, non-sterilized dentine has been left, an easy matter.

7. The fact that dentine and enamel form so exceedingly poor culture substrata for schizomycetes, is an additional proof of the position that their attack upon the teeth is only secondary; i. e., they owe their rapid development to the secretions, deposits, etc., of the oral cavity, and not until the tissue of the tooth has undergone a certain change—first decalcification, second peptonization—can they adapt it to their nourishment. The decalcification is produced chiefly by acid, resulting from the action of the organisms upon certain carbohydrates in the human mouth, while the peptonization is produced, either by the direct action of the protoplasm of the organisms upon the decalcified dentine, or by the action of a ferment which they produce.

A knowledge of the properties of the fungi of the human mouth, as given above, combined with a microscopic and chemical examination of carious tissue, and comparative studies of caries of living and dead teeth, appear to me to furnish a fair solution of the phenomena of dental caries. That other agents than those of a parasitic nature are also often concerned, there can be no doubt. To say nothing of predisposing causes, an acid reaction of the oral secretions, acid medicines, acid foods, etc., may give rise to caries at points which otherwise probably would have escaped.

## Figures and Tables

**Fig. 1 f1:**
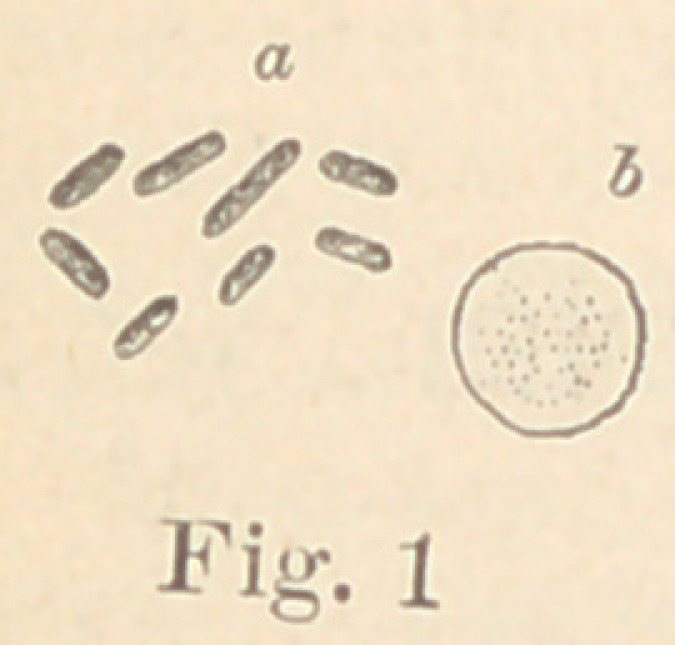


**Fig. 2 f2:**
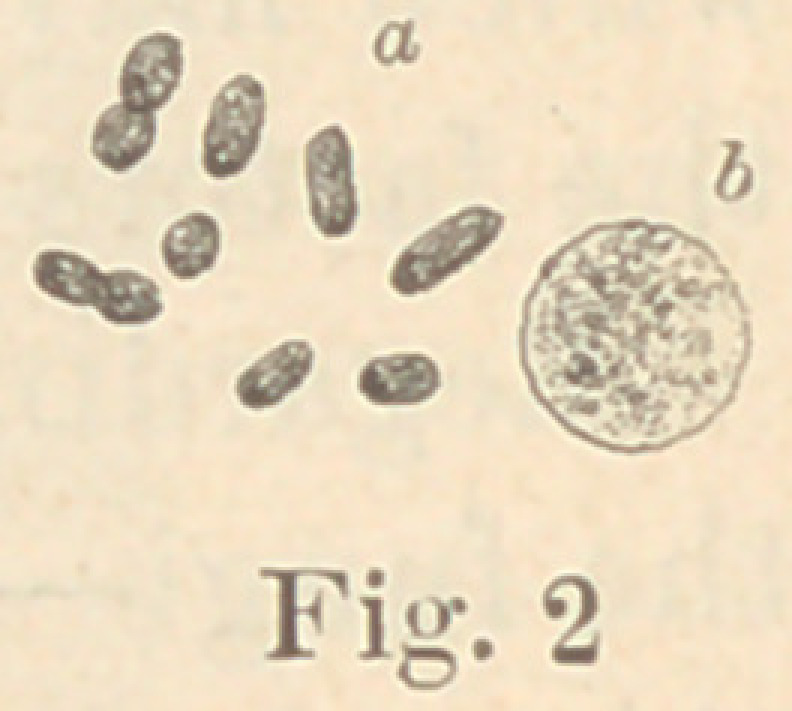


**Fig. 3 f3:**
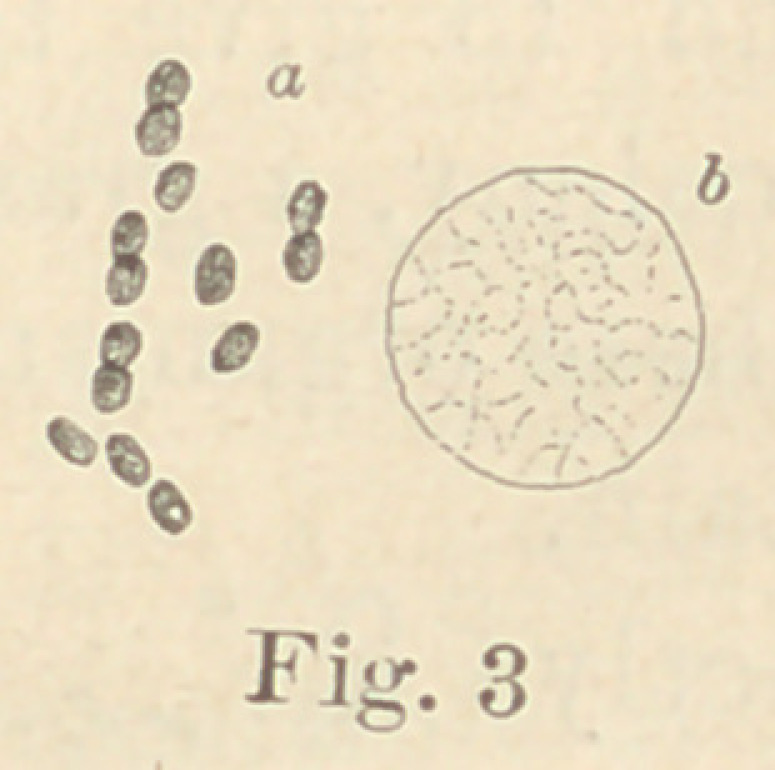


**Fig. 4 f4:**
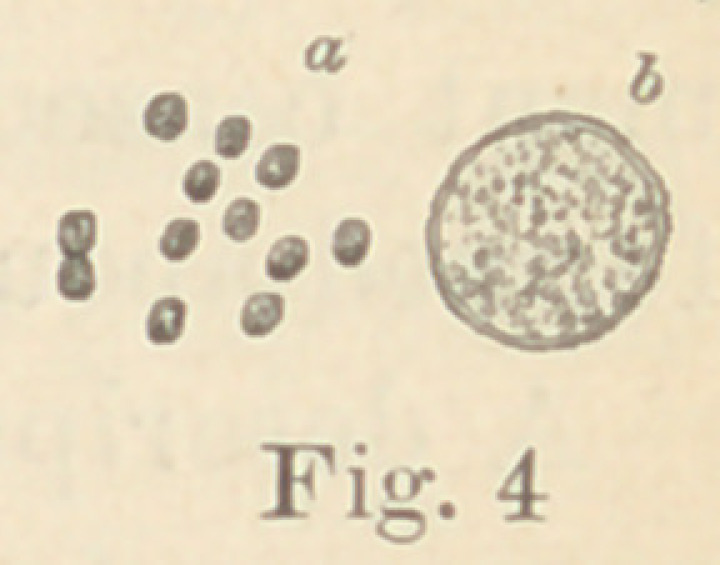


**Fig. 5 f5:**
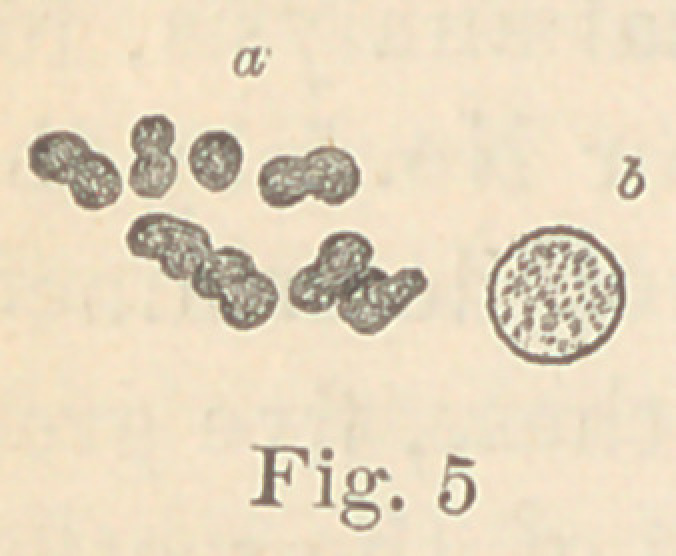


**Fig. 6 f6:**
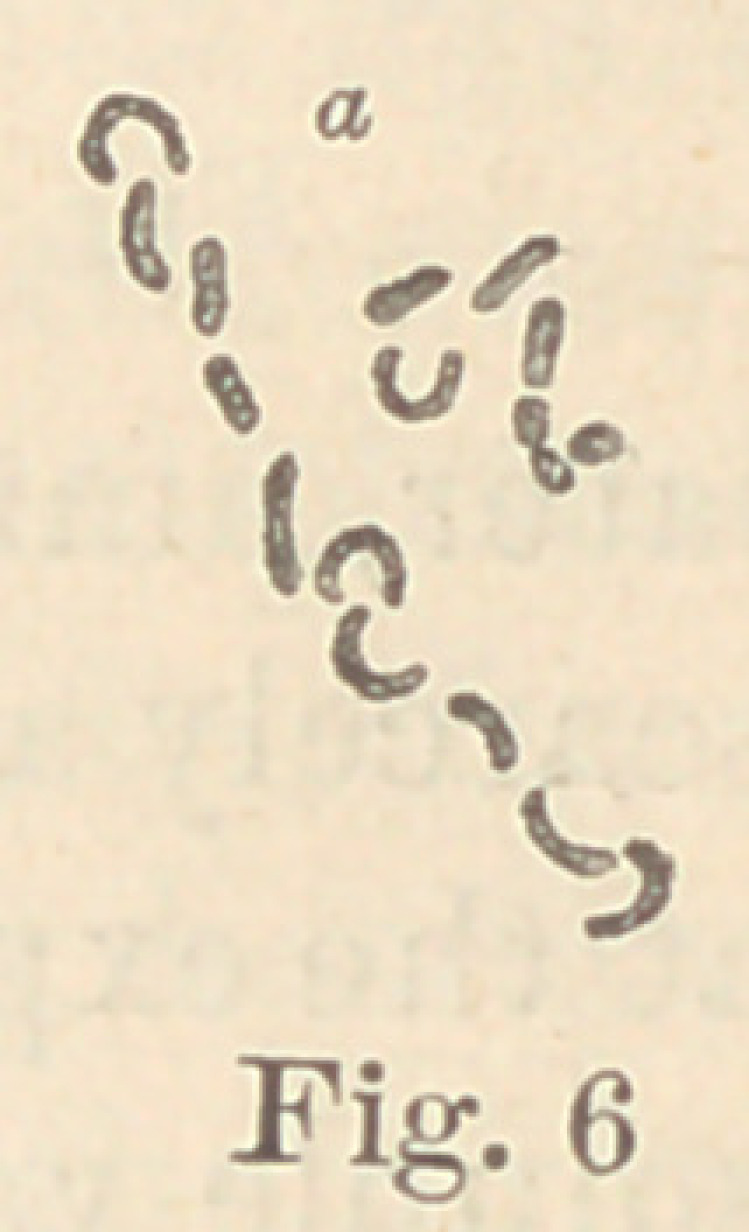


**Fig. 7 f7:**
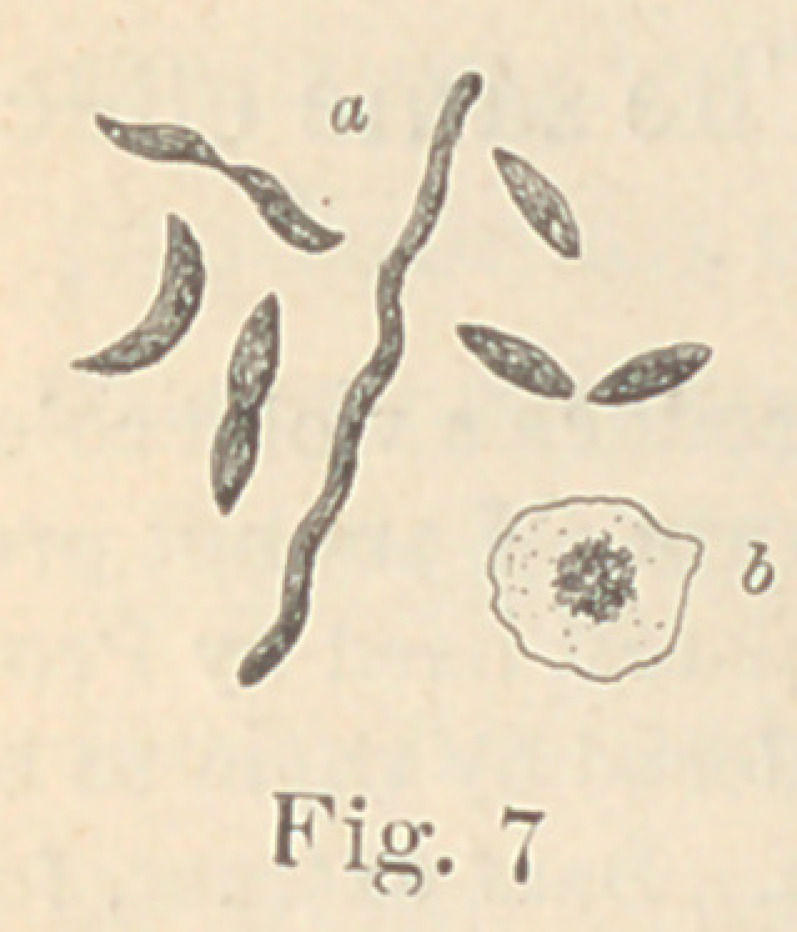


**Fig. 8 f8:**
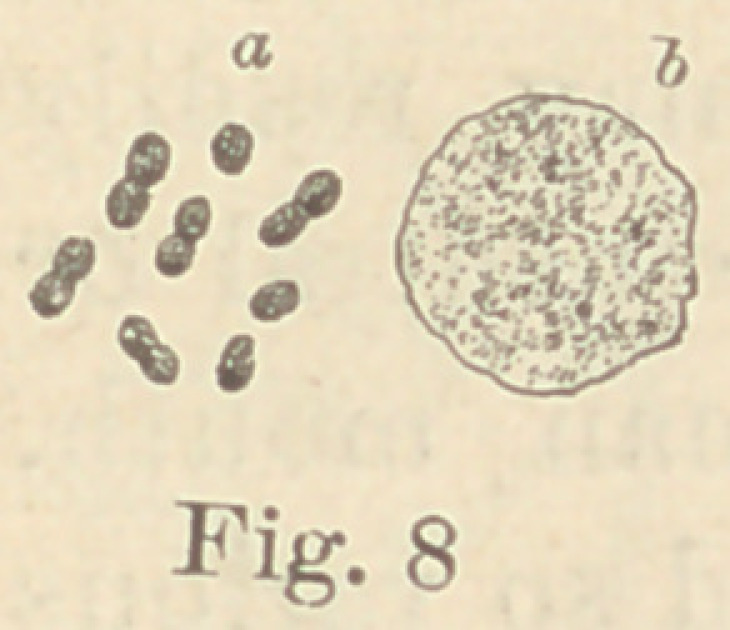


**Fig. 9 f9:**
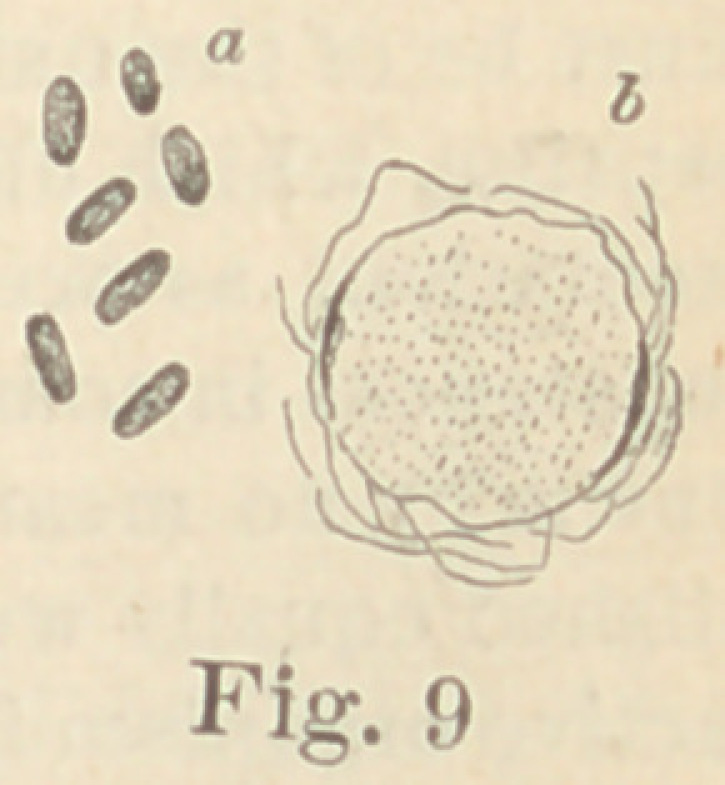


**Fig. 10 f10:**
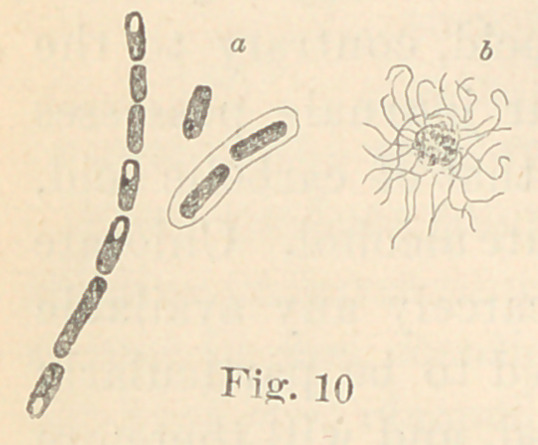


**Fig. 11 f11:**
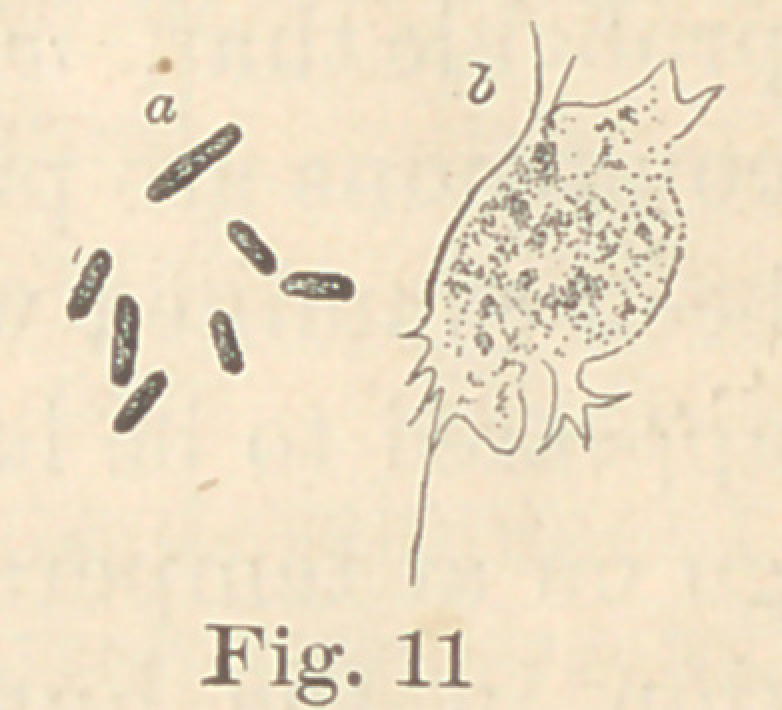


**Fig. 12 f12:**
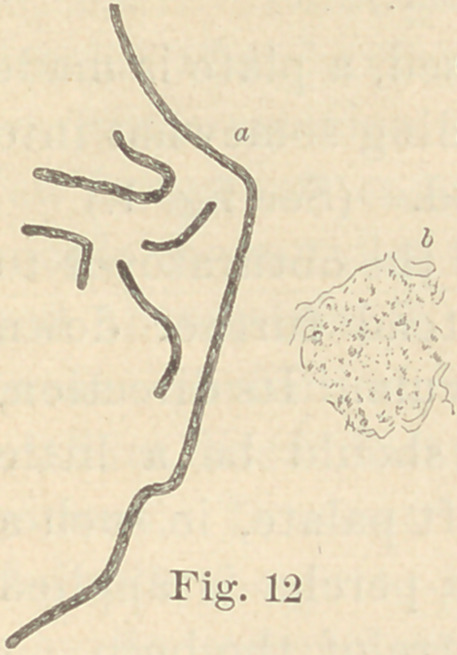


**Fig. 13 f13:**